# Supplementary figures and images for: An attenuated vaccinia vaccine encoding the severe acute respiratory syndrome coronavirus-2 spike protein elicits broad and durable immune responses, and protects cynomolgus macaques and human angiotensin-converting enzyme 2 transgenic mice from severe acute respiratory syndrome coronavirus-2 and its variants
Source: Front Microbiol. 2022 Nov 18;13:967019. doi: 10.3389/fmicb.2022.967019 (PMC9716133; doi:10.3389/fmicb.2022.967019)

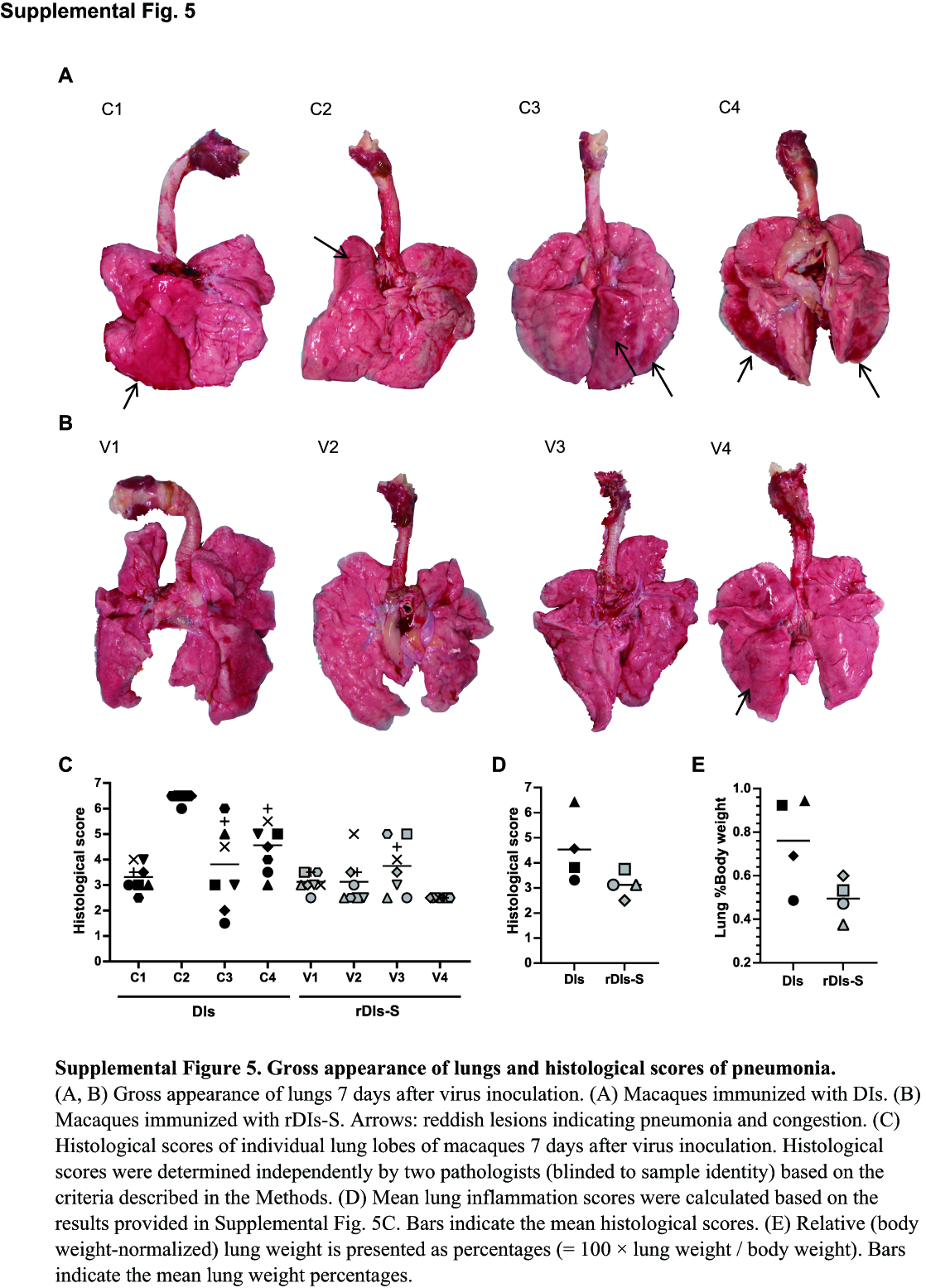

Supplement: Supplementary file 1 [file Data_Sheet_1.zip › Supplemental Fig.5.tif]

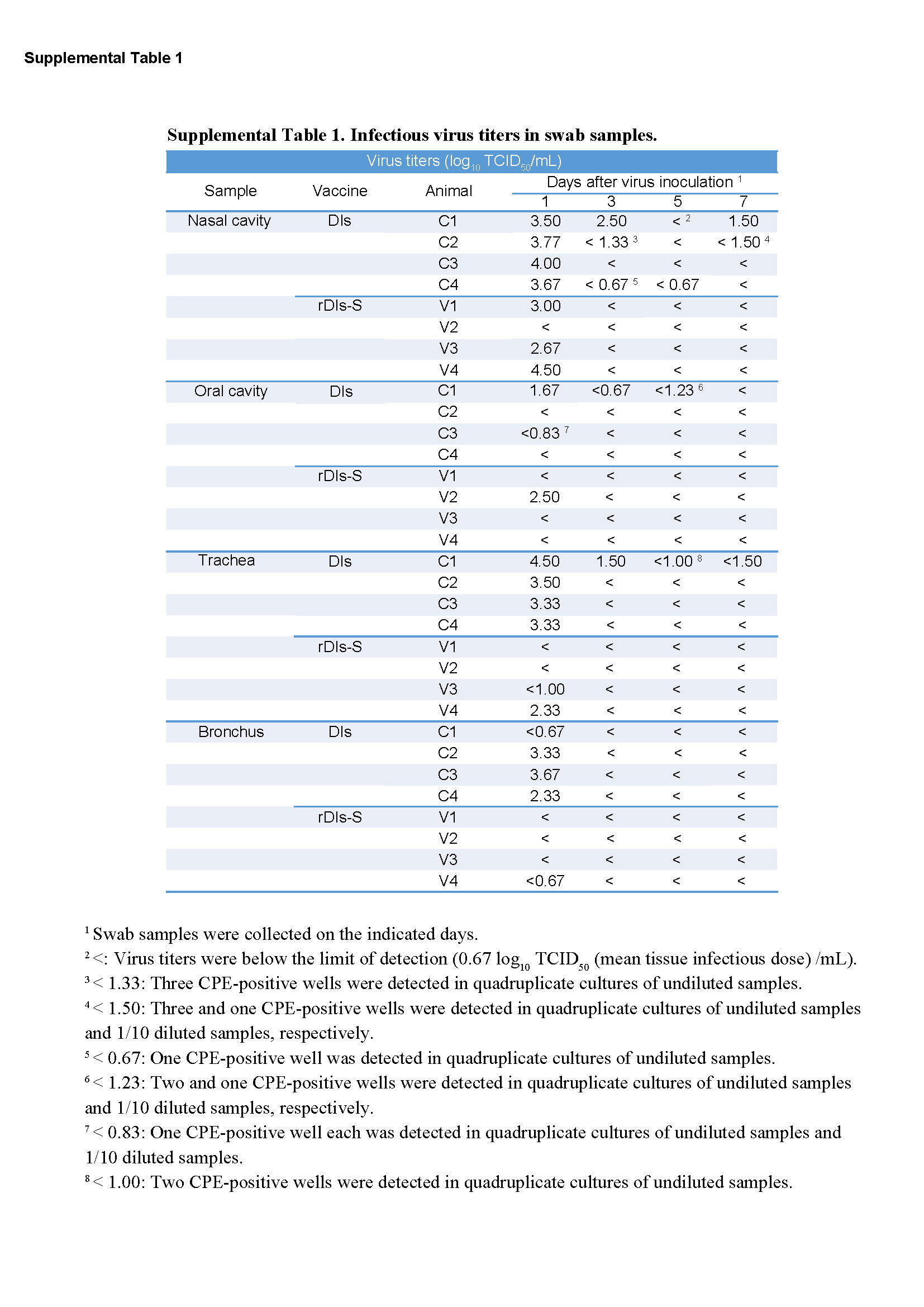

Supplement: Supplementary file 1 [file Data_Sheet_1.zip › Supplemental Table 1.TIFF]

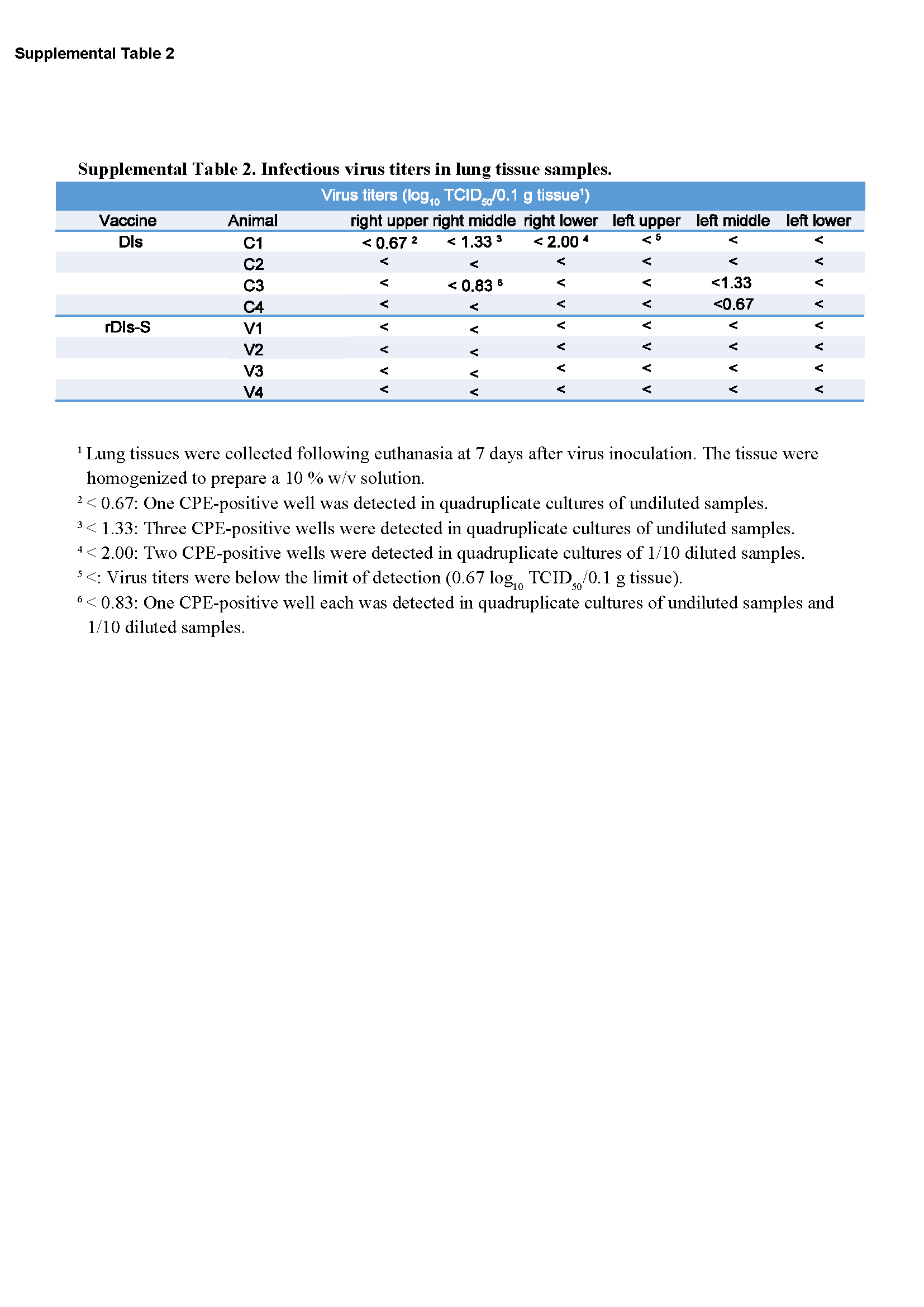

Supplement: Supplementary file 1 [file Data_Sheet_1.zip › Supplemental Table 2.TIFF]

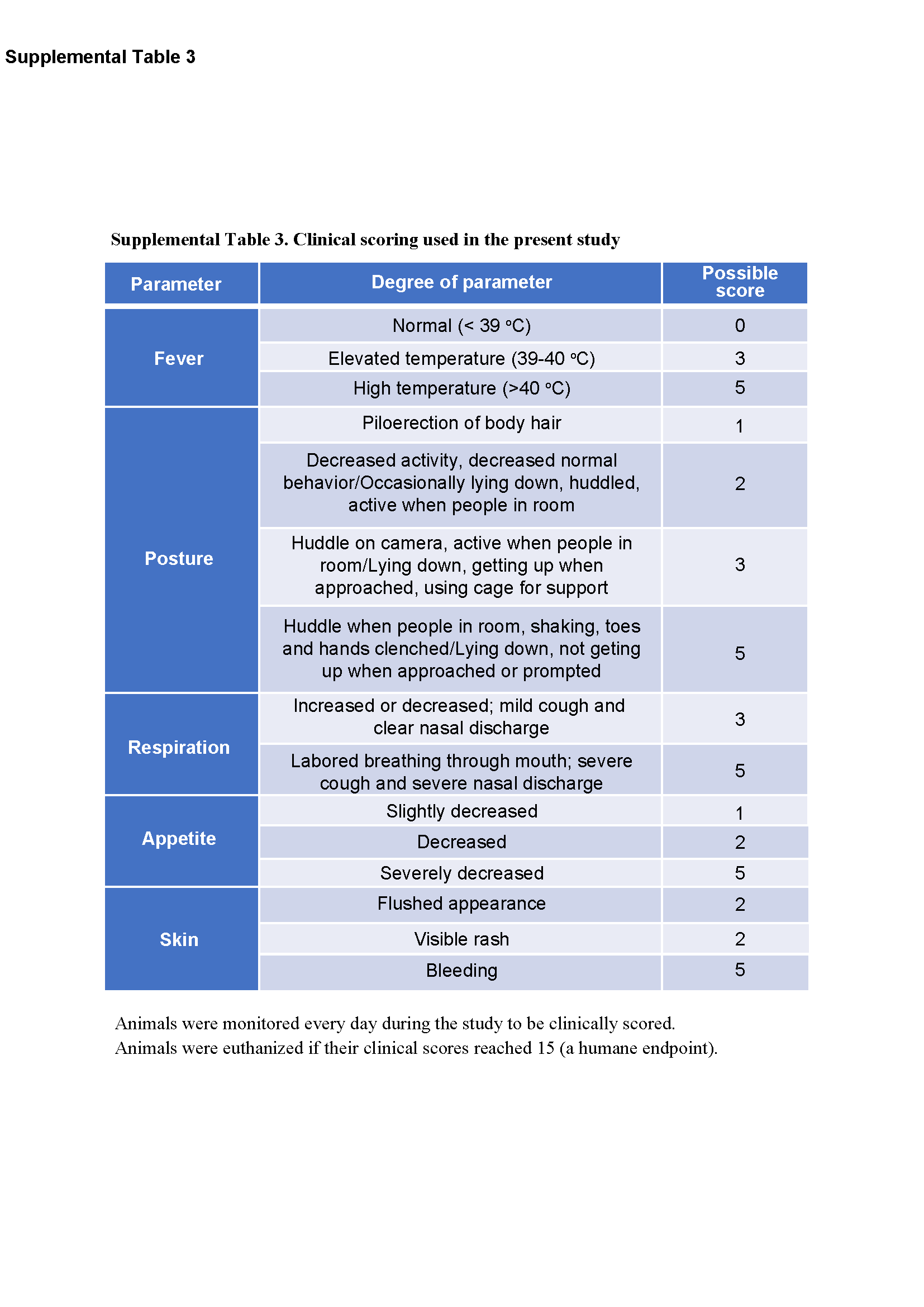

Supplement: Supplementary file 1 [file Data_Sheet_1.zip › Supplemental Table 3.TIFF]

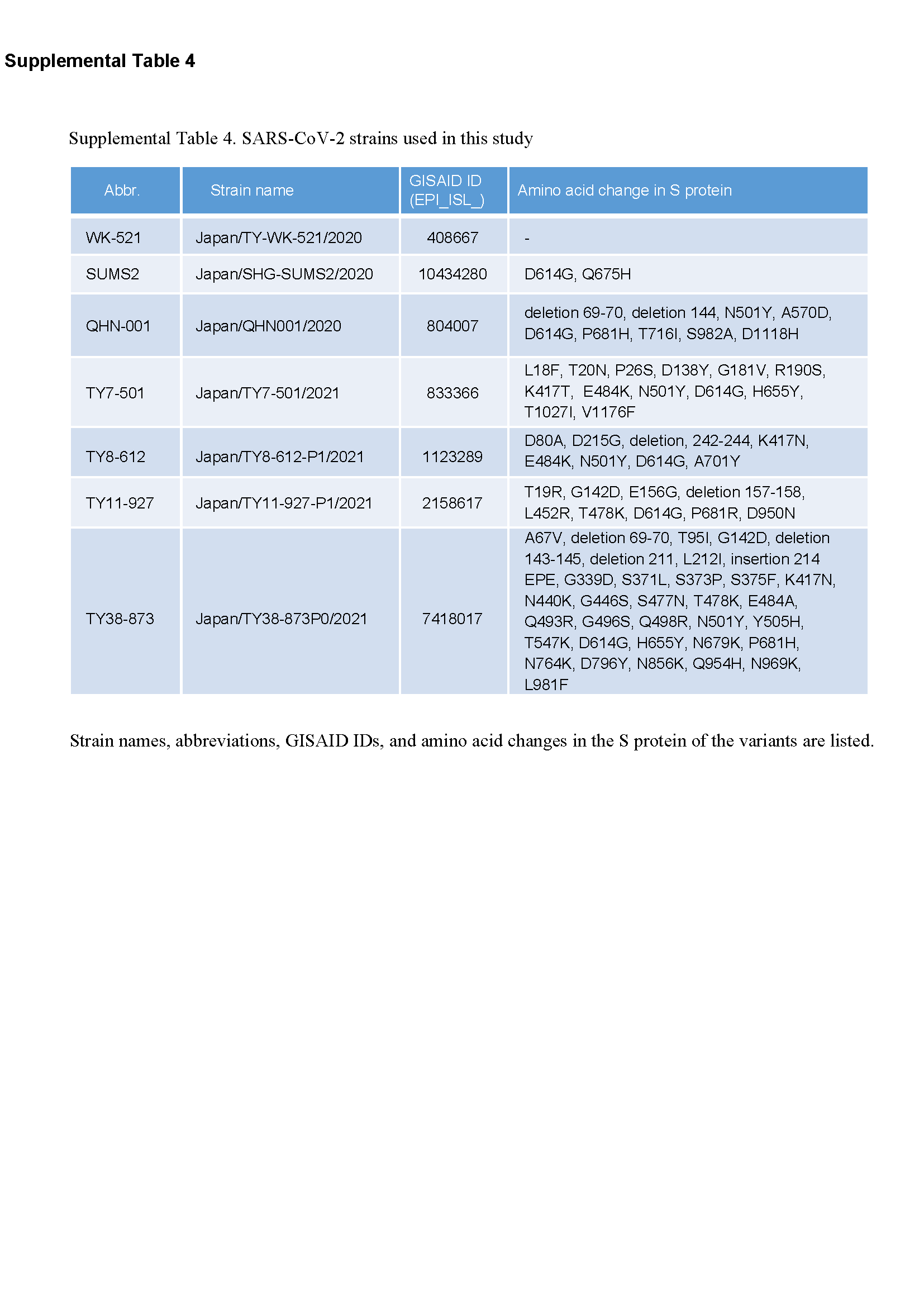

Supplement: Supplementary file 1 [file Data_Sheet_1.zip › Supplemental Table 4.TIFF]
